# Problem‐based shared decision making: The role of canonical SDM steps

**DOI:** 10.1111/hex.13654

**Published:** 2022-11-29

**Authors:** Victor M. Montori, Merel M. Ruissen, Megan E. Branda, Ian G. Hargraves, Marleen Kunneman

**Affiliations:** ^1^ Knowledge and Evaluation Research Unit Mayo Clinic Rochester Rochester Minnesota USA; ^2^ Department of Medicine, Division of Endocrinology Leiden University Medical Center Leiden Zuid‐Holland The Netherlands; ^3^ Department of Biomedical Data Sciences, Section of Medical Decision Making Leiden University Medical Center Leiden The Netherlands; ^4^ Department of Quantitative Health Sciences, Division of Clinical Trials and Biostatistics Mayo Clinic Rochester Minnesota USA

**Keywords:** problem‐based, purposeful, SDM, shared decision making, steps

## Abstract

**Objective:**

To evaluate the extent to which the canonical steps of shared decision making (SDM) take place in clinical encounters in practice and across SDM forms.

**Methods:**

We assessed 100 randomly selected video‐recorded primary care encounters, obtained as part of a randomized trial of an SDM intervention in patients with type 2 diabetes. Two coders, working independently, noted each instance of SDM, classified it as one of four problem‐based forms to SDM (weighing alternatives, negotiating conflicting issues, solving problems, or developing existential insight), and noted the occurrence and timing of each of the four canonical SDM steps: fostering choice awareness, providing information, stating preferences, and deciding. Descriptive analyses sought to determine the relative frequency of these steps across each of the four SDM forms within each encounter.

**Results:**

There were 485 SDM steps noted (mean 4.85 steps per encounter), of which providing information and stating preferences were the most common. There were 2.7 (38 steps in 14 encounters) steps per encounter observed in encounters with no discernible SDM form, 3.4 (105 steps in 31 encounters) with one SDM form, 5.2 (129 steps in 25 encounters) with two SDM forms, and 7.1 (213 steps in 30 encounters) when ≥3 SDM forms were observed within the encounter. The prescribed order of the four SDM steps was observed in, at best, 16 of the 100 encounters. Stating preferences was a common step when weighing alternatives (38%) or negotiating conflicts (59.3%) but less common when solving problems (29.2%). The distribution of SDM steps was similar to usual care with or without the SDM intervention.

**Conclusion:**

The normative steps of SDM are infrequently observed in their prescribed order regardless of whether an SDM intervention was used. Some steps are more likely in some SDM forms but no pattern of steps appears to distinguish among SDM forms.

**Clinical Trial Registration:**

ClinicalTrial.gov: NCT01293578.

## 1 INTRODUCTION

Clinical care requires noticing the problematic human situation of patients and responding with plans of care that fit. This has been defined as the work patients and clinicians do to iteratively develop a plan of care that is maximally responsive to this problematic situation, maximally supportive of patient goals, and minimally disruptive of each person's life and loves.^1^ One process by which patients and clinicians work together to figure out what to do is called shared decision making (SDM). Guidelines and other policy instruments increasingly recommend and promote the use of SDM in clinical practice.^2^, ^3^

Conventionally, SDM is framed as a decision‐making process involving patients choosing between multiple acceptable treatment options.^4^ Experts describe SDM as consisting of four consecutive steps: (1) fostering choice awareness, (2) providing information about the available options and their pros and cons, (3) deliberating about these options based on patient preferences, and (4) making a final decision.^5^, ^6^ This form of SDM is considered relatively rare in practice, its use is hampered by lack of time and other supportive resources (e.g., SDM tools), clinician's lack of ability or willingness, and other barriers.^7^

This canonical form of SDM, however, seems inappropriate as a tactic to address problems that require a method of making collaborative decisions other than weighing alternative options based on patient preferences. Recently, Hargraves and colleagues have proposed that the appropriate SDM method must purposefully match the kind of problematic situation patients and clinicians are facing.^8^

Recognizing a range of situations for which SDM is appropriate, purposeful SDM proposes four SDM forms, one for each kind of problematic situation: (1) weighing treatment alternatives, (2) negotiating intra‐, or interpersonal conflicting issues, (3) problem solving and (4) developing existential insight.^8^ After re‐analysing a database of video recordings of clinical encounters between patients with diabetes and their clinician, Ruissen et al.^9^ found that clinicians and patients frequently used SDM in practice, in 86 of 100 encounters, with the canonical SDM form of weighing treatment alternatives comprising only 33% of all purposeful SDM forms used.

After recognizing that SDM is common in the care of chronic patients and that a range of forms is used in practice, we sought to determine how often are the canonical steps of SDM seen in practice, appear in their normative order or at all within each of the forms of SDM observed. We hypothesized that the steps of SDM appear in the order prescribed when the canonical form of SDM is used (weighing treatment alternatives) but are less appropriate to describe other forms to SDM.

## 2 METHODS

We used the same data set developed for the study by Ruissen et al.^9^ for this analysis. Briefly, M.M.R. used a random‐number generator to randomly select 100 video‐recorded encounters of the 350 encounters from both arms (without stratification by arm) of a multicenter clinical trial assessing the effect of a within‐encounter SDM conversation aid (intervention) versus usual primary diabetes care for patients with type 2 diabetes in the United States (ClinicalTrial.gov: NCT01293578).^10^ The trial database was the source of patient and clinician characteristics and trial arm (usual care with or without SDM intervention) allocation.

The Mayo Clinic Institutional Review Board approved this secondary analysis before coding. Patients and clinicians provided written informed consent about the use of trial data and video recordings for research before the encounter.

Purposeful SDM provided the underpinning of the coding scheme to determine the form or forms of SDM used in an encounter.^8^ When a form of SDM was identified, a distinction was made between SDM concerning (1) weighing treatment alternatives (canonical SDM), (2) negotiating intra‐, or interpersonal issues, (3) problem solving or (4) developing existential insight. Only the start of the SDM process was coded, given the fact that a clear end of SDM can often not be distinguished. We then noted when the following conventional SDM steps appeared during the consultation: (1) fostering choice awareness, (2) providing information (including the pros/cons of available options), (3) expression of patient preference or desire, and (4) making a final decision.

We developed and refined a coding scheme based on 14 video‐recorded encounters not included in our sample. Of the 100 included videos, 20 were used to train, and test the self‐developed coding scheme. These videos and the other 80 recordings were coded using the final version of the coding scheme. All encounters were coded in duplicate by two investigators from different backgrounds (M.M.R., a medical doctor, and M.K., a clinical linguist and decision scientist). Disagreements were resolved by discussion and consensus.

### 2.1 Statistical analyses

We tested associations using the Kruskal–Wallis test for continuous variables and the *χ* ^2^ test statistic for categorical variables.

To visualize the distribution of purposeful forms and canonical steps within the encounters, we created a swimmer plot. Encounters were grouped into the plot by the number of forms present in each encounter (None, one, two, or three or more forms). The relative occurrence in time of each form noted or of each step identified is presented as the fraction of the encounter duration (i.e., from greeting to end of the visit indicated by the clinician and/or patient leaving the room or end of the recording) at which time the form or step started, expressed as a percentage of the encounter duration. Study data were collected and managed using REDCap electronic data capture tools, hosted at Mayo Clinic thanks to its Center for Clinical and Translational Science (funded by the National Institutes of Health—NCATS UL1TR002377).^11^, ^12^ Analyses were completed in SAS v9.4 (SAS, Inc.).

## 3 RESULTS

### 3.1 Participants

Table 1 describes the 100 patients (41% women, average age 60, 85% white) and 52 clinicians (28% women, average age 47) involved in the encounters included and coded. The average length of the clinical encounter was 17.0 min (range: 4.0–43.6 min).

**Table 1:** Participant characteristics

Patient characteristics	Patients (*n* = 100)
Encounter, usual care without/with SDM tool, *n*	31/69
Age (years), mean (SD)	60.0 (9.7)
Women, *n*	41
Body mass index, mean (SD)	36.7 (9.1)
Race, Black/White/other, *n*	9/85/6
Insurance, private/government/other, *n*	52/29/7
Education, high school or less, *n*	29
HbA1c, mean (SD)	8.9% (1.3)
Years in relationship with clinician, *n*	
<5	43
5 to <10	22
>10	25
Adequate health literacy, *n* a	81

Clinician characteristics	Clinicians (*n* = 52)
Age, years, mean (SD)	46.9 (11.2)
Women, *n* (%)	25 (48%)
Years in practice, mean (SD)	13.6 (10.5)
Number of encounters, mean (SD)	1.9 (1.3)
Median (IQR)	1 (1, 3)

Abbreviations: IQR, interquartile range; SDM, shared decision making.

a Based on ‘never’ or ‘rarely’ answers to the Single Item Literacy Screener (‘How often do you need to have someone help you when you read instructions, pamphlets, or other written material from your doctor or pharmacy?’).^18^

### 3.2 Purposeful forms and canonical steps of SDM

One or more SDM forms could be identified in 86 of 100 encounters. A single SDM form was evident in 31 encounters, 2 forms in 25, and 3 or more SDM forms in 30 encounters. Situations in which treatment alternatives were weighed accounted for 33% of the SDM forms used during the consultation, compared with 30% in which negotiating intra‐ or interpersonal conflicting issues was used, and 36% in which a problem‐solving form was used. Developing existential insight accounted for 1% of the observed SDM forms.

Table 2 describes the distribution of SDM steps within the encounters. In these 100 encounters, we observed 485 steps or an average of 4.85 steps per encounter. In encounters with no discernible purposeful SDM form, we observed 2.7 (38 steps in 14 encounters) steps per encounter. In encounters with one SDM form, we observed 3.4 (105 steps in 31 encounters) steps per encounter. We observed 5.2 (129 steps in 25 encounters) steps per encounter in encounters with two SDM forms and 7.1 (213 steps in 30 encounters) steps per encounter in encounters with ≥3 SDM forms observed within the encounter. The most common steps were ‘giving statements of preference or desire’ during deliberations and ‘providing information’; both steps were present in about a third of encounters with one or more purposeful SDM forms. ‘Choice awareness’ and ‘deciding’ were evident in a fifth of purposeful SDM forms.

**Table 2:** Distribution of shared decision‐making (SDM) steps and forms within encounters

	Encounters by number of SDM forms observed				
	None (*n* = 14)	One (*n* = 31)	Two (*n* = 25)	≥3 (*n* = 30)	**All encounters (*n*** = **100)**
*Steps*, *n* (%)					
SDM steps observed^a^	38^b^	105	129	213	485
Choice awareness	8 (21.1)	19 (18.1)	22 (17.1)	40 (18.8)	89 (18.4)
Providing information	13 (34.2)	32 (30.5)	39 (30.2)	53 (24.9)	137 (28.2)
Deliberating with statement of preferences	6 (15.8)	32 (30.5)	46 (35.7)	93 (43.7)	177 (36.5)
Deciding	11 (28.9)	22 (21.0)	22 (17.1)	27 (12.7)	82 (16.9)
Encounters with SDM steps in order, *n* (%)^c^	0 (0)	3 (9.7)	5 (20.0)	8 (26.7)	16 (16.0)

^a^ *χ* ^2^ test, *p* = .048.

^b^ Although no purposeful SDM was observed in these encounters, SDM steps were seen but without contributing to collaborative decision making to address the patient's problematic situation.

^c^ Fisher's exact test, *p* < .001.

When purposeful SDM was not evident, ‘giving statements of patient preference or desire’ during deliberation was less common (15.8% vs. 30.5%–43.7% when a form of purposeful SDM was observed) and ‘deciding’ (28.9% vs. 12.7%–21% when a form of purposeful SDM was observed) was more common.

SDM steps appeared in the canonical order (i.e., starting with fostering choice awareness and finishing with making a final decision) in 18 encounters. In 16 of these encounters, these sets of ordered steps were preceded or followed by other steps (Table 2). The distribution of steps within forms was similar whether the encounter was allocated to usual care with or without the SDM intervention (Appendix A).

Table 3 shows the distribution of SDM steps within each of the four forms to purposeful SDM. ‘Stating preferences’ was a common step when participants engaged in SDM by weighing treatment alternatives (38%) or negotiating intra‐interpersonal conflicts (59.3%), but less common when they worked on solving problems (29.2%) or developing an existential insight (27.3%). Appendix B shows that allocation to the SDM intervention did not affect the frequency of steps observed in total or within each SDM form. Similarly, our post hoc exploration of the duration of the care relationship (<5 vs. ≥5 years) did not affect the results (data not shown).

**Table 3:** Distribution of shared decision‐making (SDM) steps by the form of SDM in which they were observed

	SDM form^a^				
	Weighing alternatives	Negotiating conflict	Solving problems	Developing insight	**Total** b
Steps, *n* (%)					
SDM steps observed	137	108	120	11	376
Choice awareness	24 (17.5)	10 (9.3)	22 (18.3)	4 (36.4)	60 (16)
Providing information	34 (24.8)	18 (16.7)	39 (32.5)	3 (27.3)	94 (25)
Deliberating with statement of preferences	52 (38)	64 (59.3)	35 (29.2)	3 (27.3)	154 (41)
Deciding	27 (19.7)	16 (14.8)	24 (20)	1 (9.1)	68 (18.1)

^a^ *χ* ^2^ *p* value = .0011.

^b^ Data limited to encounters in which a step followed the onset of an SDM form (i.e., 83 of the 86 encounters in which an SDM form was observed).

**Figure 1:**
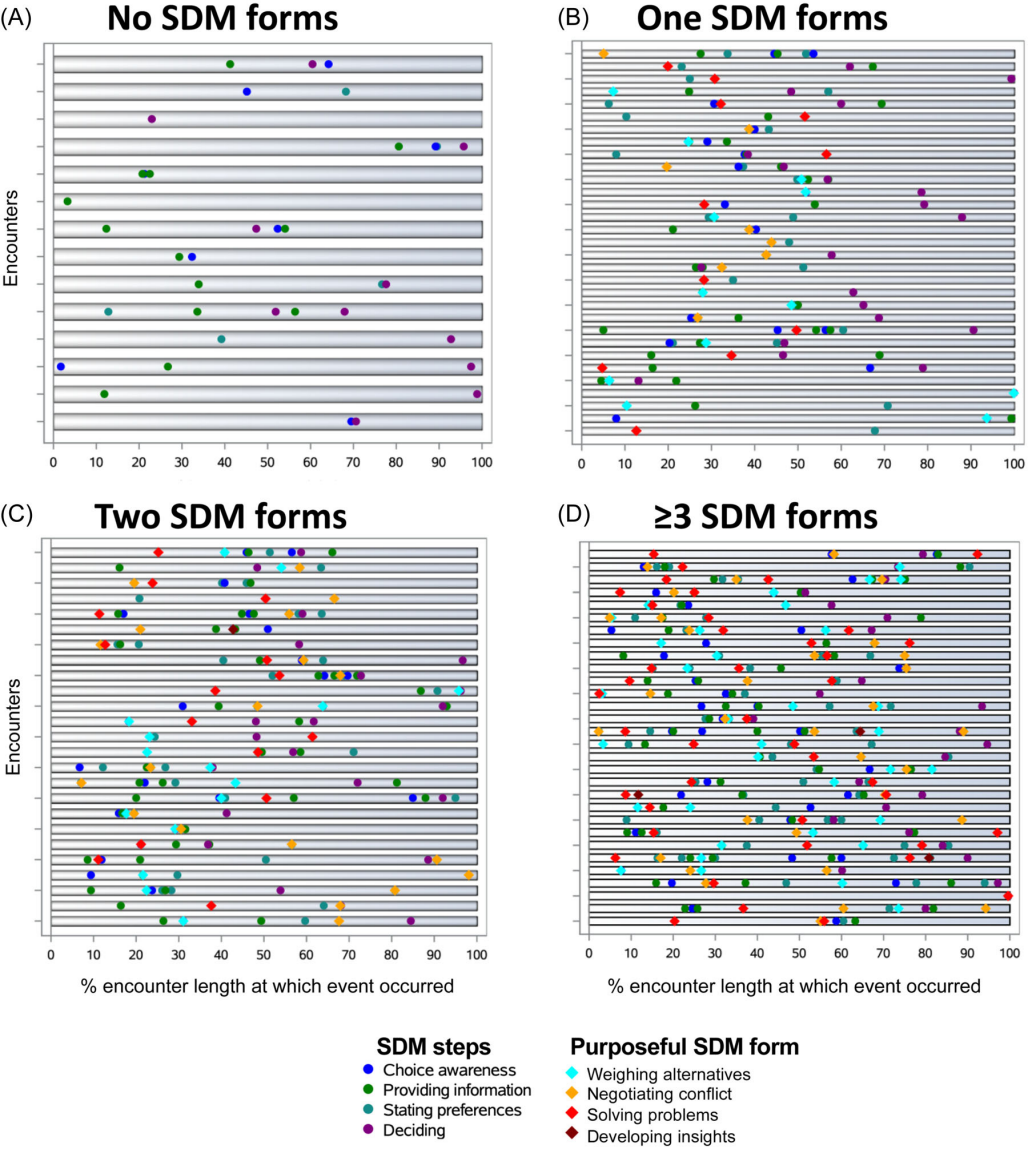
Occurrence of shared decision‐making steps and forms within encounters grouped by the number of SDM forms observed per encounter. (A) Encounters in which no shared decision‐making form was observed (*n* = 14). (B) Encounters in which one form was observed (*n* = 31). (C) Encounters in which two forms were observed (*n* = 25). (D) Encounters in which three or more forms were observed (*n* = 30). Each row represents an encounter, with its duration represented on a 100% scale.

Figure 1 describes the steps observed within SDM forms presented by whether purposeful SDM was either not observed or when 1, 2 or 3 or more forms were observed.

## 4 DISCUSSION

In this set of 100 clinical encounters obtained from a practice‐based randomized trial of usual diabetes care with or without an SDM tool, in which two‐thirds of patients with diabetes and their primary care clinicians used an SDM tool, we found that patients and clinicians engaged in SDM without necessarily completing the canonical SDM steps or following them in their prescribed order. We found that the canonical steps of SDM were present when no specific purposeful SDM form was identified. These steps also were commonly present when one or more purposeful SDM forms were used (of which the canonical form of SDM represented about a third), were similarly present regardless of which SDM form was used, and were present in the normative order in, at best, 16% of encounters. In 70% of encounters, clinicians and patients took different SDM steps as they entered and switched across different forms to SDM. These results suggest that, even under stimulated conditions of adding an SDM intervention, clinicians and patients infrequently follow the normative order of SDM steps to make decisions with patients in practice.

Along with the report by Ruissen et al.,^9^ which found that almost 90% of these encounters demonstrated some form of SDM (with the canonical form representing about a third of the observed instances), this report documents the relative frequency of SDM steps in these encounters and the timing of their appearance within each encounter. The results are not directly comparable to other studies in which the frequency of steps has been analysed as if each encounter had only one form of SDM. Kunneman et al., for example, documented that choice awareness appeared in 53% of clinical encounters drawn from a similar sample of video‐recorded encounters within clinical trials of SDM tools.^13^

The results call into question SDM measurement forms that rely on the presence of SDM steps to determine the occurrence or quality of SDM.^14^, ^15^, ^16^ SDM steps occurred, in one instance in the normative order, even when no purposeful SDM form was evident. The most assessed step of SDM, providing information,^15^ appears in less than a third of instances of SDM.

These results, while novel, have limitations. Video recordings were randomly drawn from a set of encounters produced during the experimental evaluation of the use of an SDM intervention. The presence of the conversation aid, the video recorder, or of the randomized trial procedures may have affected the observations reported herein. We intuit that the direction of effect of these factors would have been to normalize the encounters to what is expected (i.e., a higher prevalence of the canonical form of SDM with the steps in the expected order). That, despite these factors, we found high variability in the range of purposeful SDM forms and canonical steps may thus represent a best‐case scenario. These findings must be evaluated in independent data sets by other research groups. On the other hand, the carefully developed yet ad‐hoc coding scheme based in part on purposeful SDM and its use by a clinician and an expert in SDM on actual clinical encounters across multiple primary care practices represent the strengths of this investigation.

These results, particularly the patterns observed in Figure 1, suggest a highly variable approach to SDM in primary care practice. This variability could be an indication of poor participant skill, or that the SDM intervention, present in two‐thirds of visits, provided insufficient support in structuring the encounter. Alternatively, this variability could represent the natural process of trial‐and‐error, of uncovering how might a problem be addressed, that patients and clinicians use during consultations.

The most common depiction of SDM, by Charles et al.,^6^ refers to stages (information exchange, deliberation, decision making) in which each one leads to the next. The Three Talk Model by Elwyn et al.^17^ suggests, instead, a cyclical process by which patient and clinician move along the steps of SDM, a process that may very well describe the observations here, particularly those within the canonical form of SDM (weighing alternatives). Both models assume that a problem is defined at the start of the process and that the exchange focuses on how to solve it.

Conversely, a major advantage of the purposeful SDM framework is the recognition that the nature of the problem and of how to respond to it can emerge from the joint effort of clinician and patient.^8^ This view matches better with the observations reported here of multiple forms to SDM and multiple steps taken as the patient and clinician talk, think, and feel their way through the uncertain and problematic human situation of the patient. The variability observed may in fact suggest flexibility in the use of clinical skills within a participatory and empathic collaboration. This possibility may need to be explored using content analysis of the encounters.

These findings, if confirmed, would give credence to the purposeful SDM model and challenge ways of training, measuring, and assessing for SDM that rely on (a) a single canonical form of SDM, and (b) a set order of steps to do SDM well. This challenge may lead to new SDM tools designed to create the conditions for flexible collaboration, supporting whichever form appears more conducive to addressing the problematic situation of the patient.

Our findings may also challenge the notion that the key problem SDM addresses is patient participation when it seems as if both patient and clinician must take part in determining together what the problem is and how to address it in an iterative and, to the outside observer, somewhat chaotic process of exploration, discovery, and experimentation.

Finally, our findings challenge existing measures of the occurrence and quality of SDM that rely on detecting only one form of SDM and one set of steps.^14^ Indeed, when clinicians say ‘but I do SDM already’ they may be referring to the processes depicted here, which depart in important ways from what has counted as SDM hitherto.

In conclusion, we found that the canonical steps of SDM are infrequently observed in their normative order in usual clinical practice (as observed in a practice‐based randomized trial of adding or not an SDM intervention), regardless of whether an SDM tool was used. These steps do not appear more likely to follow a particular order when one or more SDM forms are used within a clinical encounter. The most common steps are for patients to state their preferences or desires during deliberation and for clinicians to share information. These observations should be considered when developing new measures of SDM and interventions—for example, training and tools—to promote its optimal and purposeful use as a method of care in practice.

## AUTHOR CONTRIBUTIONS

Victor M. Montori, is the principal investigator at the KER Unit, the research entity within Mayo Clinic that conducted the original trial from which the videos for this secondary analysis came and that maintains the database with such videos. He wrote the first draft of the manuscript with Merel M. Ruissen and coordinated revisions and final submission. Merel M. Ruissen worked with Marleen Kunneman under her supervision to develop the study protocol and the video coding scheme. She conducted with Marleen Kunneman the data collection, and video analysis, and contributed to the data analyses reported here. She worked with Victor M. Montori on the first draft of this manuscript, provided critical revisions and approved the final manuscript. Megan E. Branda is the study statistician and contributes to maintaining the database of video recordings and clinical data from KER Unit trials. She worked with the co‐authors in designing and conducting the data analyses and produced the figure and tables that accompany this manuscript. She also provided critical revisions and approved the final manuscript. Ian G. Hargraves is the proponent of the purposeful shared decision‐making framework that guides this work. He worked with Merel M. Ruissen and Marleen Kunneman providing feedback on the video coding scheme and on analysing the results. Ian G. Hargraves provided critical revisions to the manuscript and approved the final submission. Marleen Kunneman worked with and provided direct supervision to Merel M. Ruissen in the development of the study protocol, coding scheme, video analyses, and data analyses and interpretation. She provided critical revisions to the manuscript and approved the final version. She is the corresponding author and data warrantor.

## CONFLICT OF INTEREST

The authors declare no conflict of interest.

## ETHICS STATEMENT

The Mayo Clinic Institutional Review Board approved this secondary analysis before coding. Patients and clinicians provided written informed consent about the use of trial data and video recordings for research before the encounter.

## Data Availability

The data extracted from video recordings that support the findings of this study are available from the corresponding author upon reasonable request.

## APPENDIX A

See Table A1

**Table A1:** Table of shared decision‐making (SDM) steps observed classified by (a) whether the encounter was allocated to usual care with or without the use of an SDM intervention, and (b) number of SDM forms observed.

	SDM forms observed per encounter					
Steps, *n* (%)	No SDM form	One form	Two forms	Three or more forms	All	*p* Value
*SDM intervention*	*N* = 23	*N* = 64	*N* = 89	*N* = 172	*N* = 348	.040^a^
Choice awareness	4 (17.4)	10 (15.6)	14 (15.7)	33 (19.2)	61 (17.5)	
Providing information	8 (34.8)	20 (31.3)	28 (31.5)	42 (24.4)	98 (28.2)	
Deliberating with statement of preferences	3 (13.0)	19 (29.7)	30 (33.7)	76 (44.2)	128 (36.8)	
Deciding	8 (34.8)	15 (23.4)	17 (19.1)	21 (12.2)	61 (17.5)	
*Usual care*	*N* = 15	*N* = 41	*N* = 40	*N* = 41	*N* = 137	.96^a^
Choice awareness	4 (26.7)	9 (22.0)	8 (20.0)	7 (17.1)	28 (20.4)	
Providing information	5 (33.3)	12 (29.3)	11 (27.5)	11 (26.8)	39 (28.5)	
Deliberating with statement of preferences	3 (20.0)	13 (31.7)	16 (40.0)	17 (41.5)	49 (35.8)	
Deciding	3 (20.0)	7 (17.1)	5 (12.5)	6 (14.6)	21 (15.3)	

^a^ *χ* ^2^ *p* value.

## APPENDIX B

See Table B1

**Table B1:** of shared decision‐making (SDM) steps observed classified by (a) whether the encounter was allocated to usual care with or without the use of an SDM intervention, and (b) by the SDM form within which the step was observed.

	SDM form^a^				
SDM steps within a form, *n* (%)	Weighing alternatives	Negotiating conflict	Solving problems	Developing insight	Total
*SDM intervention* a	*N* = 106	*N* = 69	*N* = 88	*N* = 8	*N* = 271
Choice awareness	18 (17.0)	5 (7.2)	14 (15.9)	2 (25.0)	39 (14.4)
Providing information	23 (21.7)	11 (15.9)	28 (31.8)	2 (25.0)	64 (23.6)
Deliberating with statement of preferences	43 (40.6)	42 (60.9)	28 (31.8)	3 (37.5)	116 (42.8)
Deciding	22 (20.8)	11 (15.9)	18 (20.5)	1 (12.5)	52 (19.2)
*Usual care* b	*N* = 31	*N* = 39	*N* = 32	*N* = 3	*N* = 105
Choice awareness	6 (19.4)	5 (12.8)	8 (25.0)	2 (66.7)	21 (20.0)
Providing information	11 (35.5)	7 (17.9)	11 (34.4)	1 (33.3)	30 (28.6)
Deliberating with statement of preferences	9 (29.0)	22 (56.4)	7 (21.9)	0 (0.0)	38 (36.2)
Deciding	5 (16.1)	5 (12.8)	6 (18.8)	0 (0.0)	16 (15.2)

^a^ *χ* ^2^ *p* value = .052.

^b^ *χ* ^2^ *p* value = .072.
